# Supplementary material for: Immunity in the Progeroid Model of Cockayne Syndrome: Biomarkers of Pathological Aging
Source: Cells. 2024 Feb 26;13(5):402. doi: 10.3390/cells13050402 (PMC10930946; doi:10.3390/cells13050402)
Supplement: Supplementary file 1 [file cells-13-00402-s001.zip › cells-2819006-supplementary.pdf]

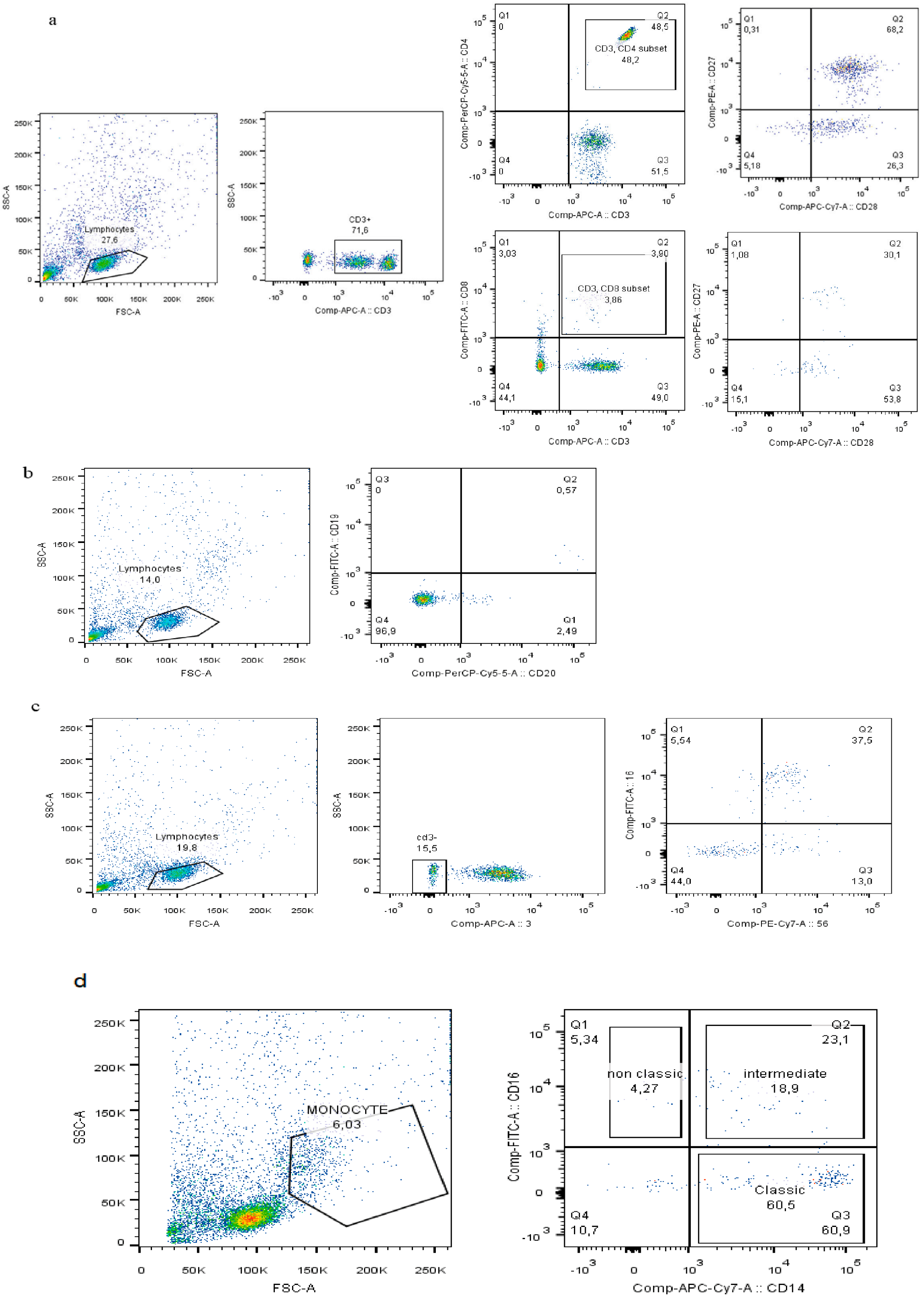

Figure S1. Gating strategy for the cell populations of the immune system based on the differential expression of different surface markers. (a) CD4+/CD8+ LT subtypes; (b) LB; (c) NK; (d) and monocyte subtypes. For each sample, 10.000 event were used.
